# Supplementary material for: T Cell-Mediated Tumor Killing-Related Classification of the Immune Microenvironment and Prognosis Prediction of Lung Adenocarcinoma
Source: J Clin Med. 2022 Dec 5;11(23):7223. doi: 10.3390/jcm11237223 (PMC9739876; doi:10.3390/jcm11237223)
Supplement: Supplementary file 1 [file jcm-11-07223-s001.zip › Table S1.docx]

Table S1. Baseline clinical characteristics of the two clustered subgroups.

| Characteristics | C1 (N=261) | C2 (N=222) | Total (N=483) | *P*-value |
| --- | --- | --- | --- | --- |
| Age | 67 (33, 87) | 65 (38, 88) | 66 (33, 88) | < 0.01 |
| Sex |  |  |  | < 0.01 |
| Female | 167 (34.58%) | 93 (19.25%) | 260 (53.83%) |  |
| Male | 94 (19.46%) | 129 (26.71%) | 223 (46.17%) |  |
| Race |  |  |  | 0.03 |
| White | 216 (44.72%) | 163 (33.75%) | 379 (78.47%) |  |
| Black | 24 (4.97%) | 25 (5.18%) | 49 (10.14%) |  |
| Asian | 1 (0.21%) | 6 (1.24%) | 7 (1.45%) |  |
| American Indian | 1 (0.21%) | 0 (0%) | 1 (0.21%) |  |
| Unknown | 19 (3.93%) | 28 (5.80%) | 47 (9.73%) |  |
| Location |  |  |  | 0.95 |
| Left | 102 (21.12%) | 85 (17.60%) | 187 (38.72%) |  |
| Right | 151 (31.26%) | 131 (27.12%) | 282 (58.39%) |  |
| Unknown | 8 (1.66%) | 6 (1.24%) | 14 (2.90%) |  |
| T |  |  |  | < 0.01 |
| T1 | 111 (22.98%) | 54 (11.18%) | 165 (34.16%) |  |
| T2 | 119 (24.64%) | 134 (27.74%) | 253 (52.38%) |  |
| T3 | 24 (4.97%) | 20 (4.14%) | 44 (9.11%) |  |
| T4 | 7 (1.45%) | 11 (2.28%) | 18 (3.73%) |  |
| Tx | 0 (0%) | 3 (0.62%) | 3 (0.62%) |  |
| N |  |  |  | < 0.01 |
| N0 | 190 (39.34%) | 122 (25.26%) | 312 (64.60%) |  |
| N1 | 40 (8.28%) | 50 (10.35%) | 90 (18.63%) |  |
| N2 | 25 (5.18%) | 44 (9.11%) | 69 (14.29%) |  |
| N3 | 1 (0.21%) | 1 (0.21%) | 2 (0.41%) |  |
| Nx | 5 (1.04%) | 5 (1.04%) | 10 (2.07%) |  |
| M |  |  |  | 0.1 |
| M0 | 167 (34.58%) | 152 (31.47%) | 319 (66.05%) |  |
| M1 | 10 (2.07%) | 15 (3.11%) | 25 (5.18%) |  |
| Mx | 84 (17.39%) | 55 (11.39%) | 139 (28.78%) |  |
| Stage |  |  |  | < 0.01 |
| Stage I | 166 (34.37%) | 95 (19.67%) | 261 (54.04%) |  |
| Stage II | 56 (11.59%) | 61 (12.63%) | 117 (24.22%) |  |
| Stage III | 29 (6.00%) | 50 (10.35%) | 79 (16.36%) |  |
| Stage IV | 10 (2.07%) | 16 (3.31%) | 26 (5.38%) |  |
| EGFR mutation |  |  |  | 0.06 |
| Yes | 35 (7.25%) | 43 (8.90%) | 78 (16.15%) |  |
| No | 111 (22.98%) | 74 (15.32%) | 185 (38.30%) |  |
| Unknown | 115 (23.81%) | 105 (21.74%) | 220 (45.55%) |  |
| KRAS mutation |  |  |  | 0.18 |
| Yes | 35 (7.25%) | 26 (5.38%) | 61 (12.63%) |  |
| No | 137 (28.36%) | 102 (21.12%) | 239 (49.48%) |  |
| Unknown | 89 (18.43%) | 94 (19.46%) | 183 (37.89%) |  |
