# Supplementary material for: T Cell-Mediated Tumor Killing-Related Classification of the Immune Microenvironment and Prognosis Prediction of Lung Adenocarcinoma
Source: J Clin Med. 2022 Dec 5;11(23):7223. doi: 10.3390/jcm11237223 (PMC9739876; doi:10.3390/jcm11237223)
Supplement: Supplementary file 1 [file jcm-11-07223-s001.zip › Table S2.docx]

Table S2. Univariate Cox regression analysis for 26 GSTTK

| Gene | Univariate Cox Regression Analysis | | *P*-value | | |
| --- | --- | --- | --- | --- | --- |
|  | Hazard Ratio | 95% Confidence Interval | Likelihood test | Logrank test | Wald test |
| BARHL2 | 2.122 | 1.128-3.994 | 0.076 | 0.018 | 0.020 |
| OIP5 | 1.440 | 1.216-1.707 | <0.001 | <0.001 | <0.001 |
| KIF20B | 1.420 | 1.138-1.772 | 0.002 | 0.002 | 0.002 |
| RAET1L | 1.419 | 1.08-1.864 | 0.027 | 0.011 | 0.012 |
| SKA3 | 1.406 | 1.176-1.679 | <0.001 | <0.001 | <0.001 |
| MAD2L1 | 1.370 | 1.162-1.616 | <0.001 | <0.001 | <0.001 |
| INCENP | 1.341 | 1.102-1.631 | 0.004 | 0.003 | 0.003 |
| KIF11 | 1.332 | 1.142-1.555 | <0.001 | <0.001 | <0.001 |
| FOXM1 | 1.309 | 1.154-1.485 | <0.001 | <0.001 | <0.001 |
| MCM10 | 1.287 | 1.081-1.531 | 0.005 | 0.004 | 0.004 |
| AURKA | 1.281 | 1.112-1.476 | 0.001 | 0.001 | 0.001 |
| CENPF | 1.265 | 1.103-1.451 | 0.001 | 0.001 | 0.001 |
| AURKB | 1.202 | 1.062-1.359 | 0.004 | 0.003 | 0.003 |
| PMAIP1 | 1.149 | 1.014-1.301 | 0.030 | 0.029 | 0.029 |
| SFTPA2 | 0.947 | 0.906-0.99 | 0.018 | 0.016 | 0.016 |
| HSD17B13 | 0.814 | 0.671-0.986 | 0.020 | 0.034 | 0.035 |
| CR2 | 0.801 | 0.692-0.927 | 0.002 | 0.003 | 0.003 |
| MICALCL | 0.795 | 0.636-0.993 | 0.040 | 0.043 | 0.043 |
| SULT1A1 | 0.761 | 0.615-0.941 | 0.010 | 0.012 | 0.012 |
| DNASE1L3 | 0.695 | 0.533-0.906 | 0.004 | 0.007 | 0.007 |
| KCNRG | 0.646 | 0.442-0.944 | 0.012 | 0.023 | 0.024 |
| TLR10 | 0.645 | 0.498-0.836 | <0.001 | 0.001 | 0.001 |
| AVPR2 | 0.559 | 0.326-0.96 | 0.022 | 0.036 | 0.035 |
| CLECL1 | 0.501 | 0.344-0.731 | <0.001 | <0.001 | <0.001 |
| GLIPR1L2 | 0.405 | 0.175-0.936 | 0.028 | 0.034 | 0.035 |
| MYF6 | 0.005 | 0-0.34 | 0.005 | 0.013 | 0.014 |
