# Supplementary figures and images for: T Cell-Mediated Tumor Killing-Related Classification of the Immune Microenvironment and Prognosis Prediction of Lung Adenocarcinoma
Source: J Clin Med. 2022 Dec 5;11(23):7223. doi: 10.3390/jcm11237223 (PMC9739876; doi:10.3390/jcm11237223)

A

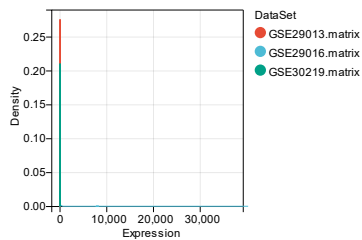

B

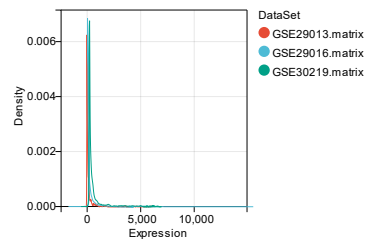

C

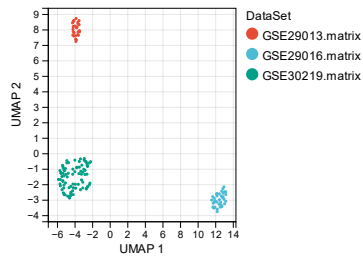

D

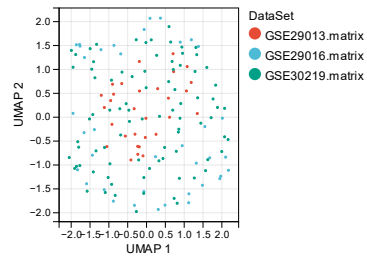

E

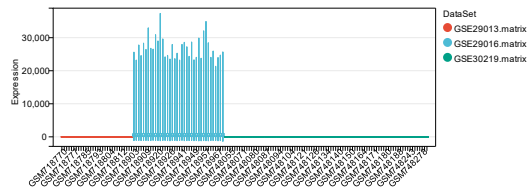

F

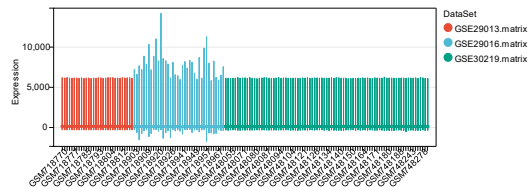

Supplement: Supplementary file 1 [file jcm-11-07223-s001.zip › Figure S1.pdf]

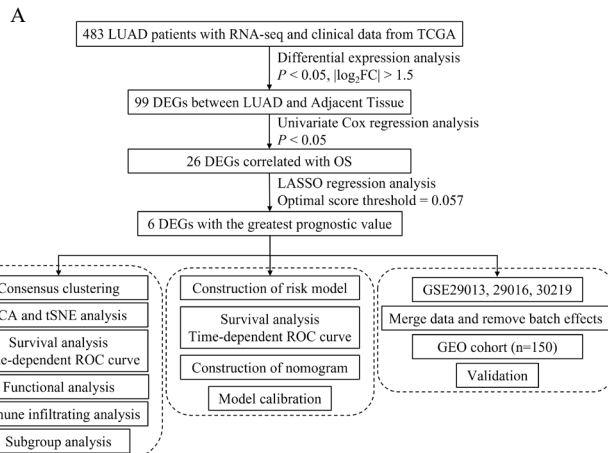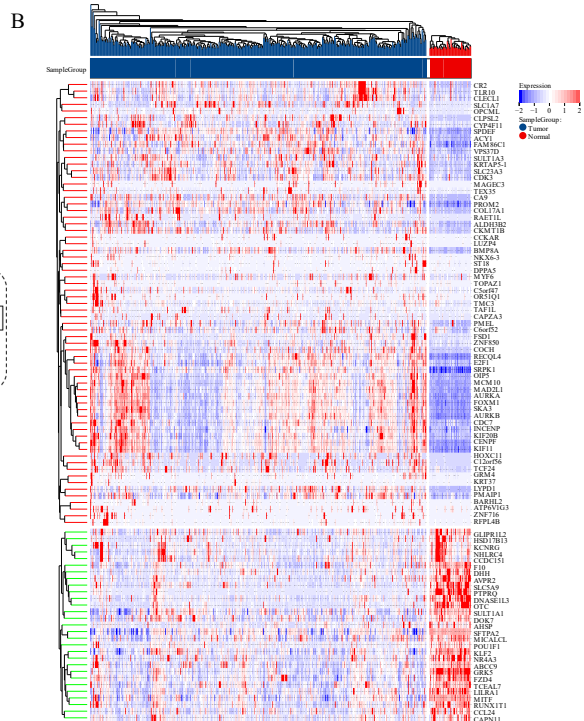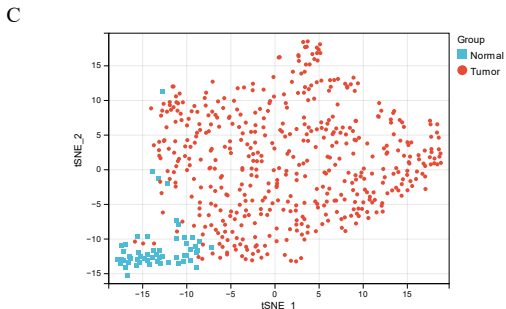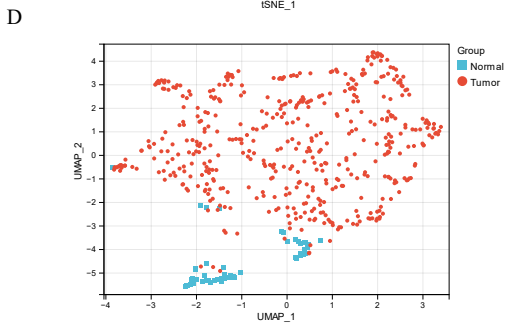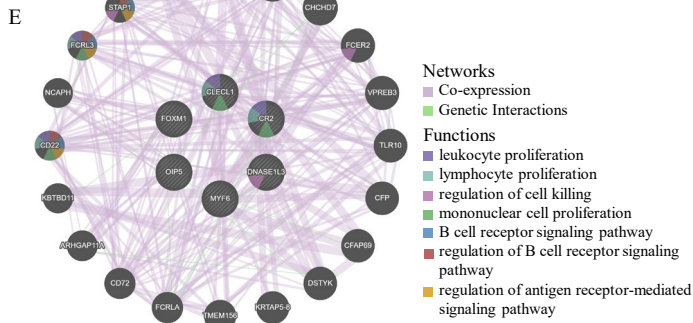

Supplement: Supplementary file 1 [file jcm-11-07223-s001.zip › Figure S2.pdf]

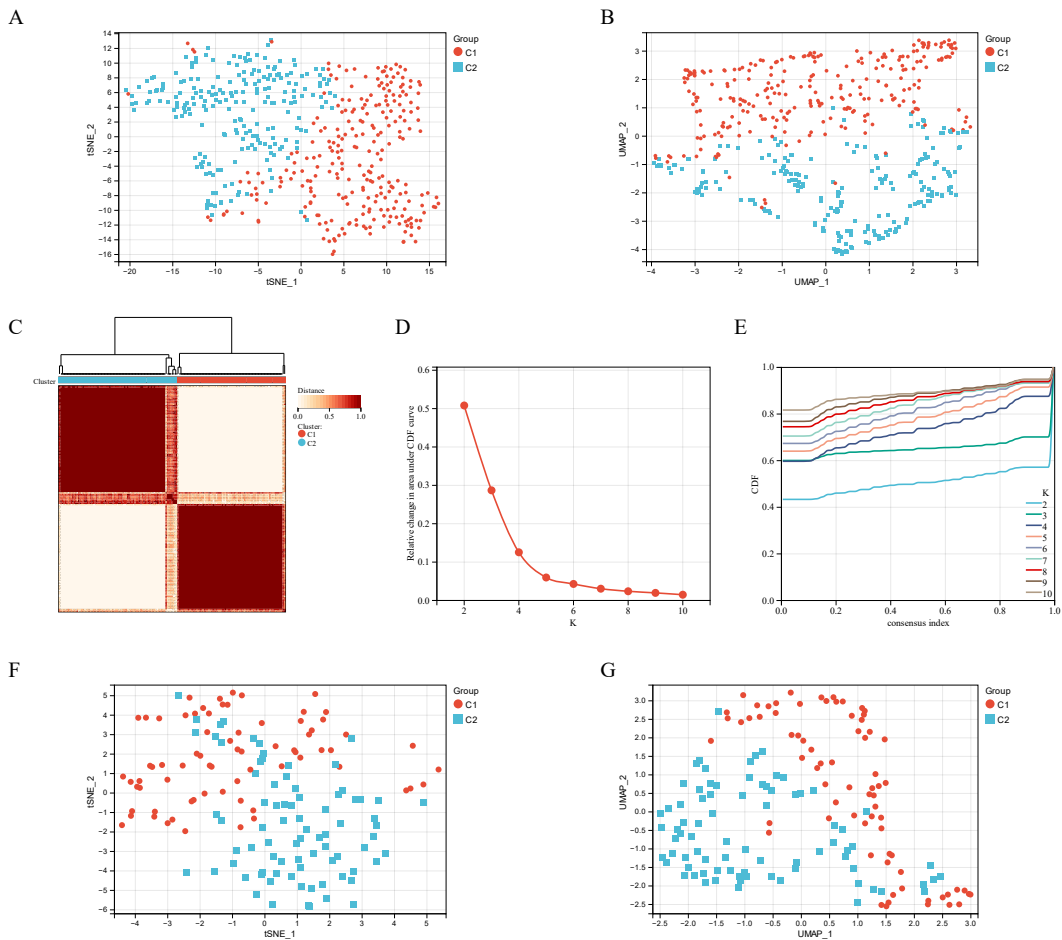

Supplement: Supplementary file 1 [file jcm-11-07223-s001.zip › Figure S3.pdf]

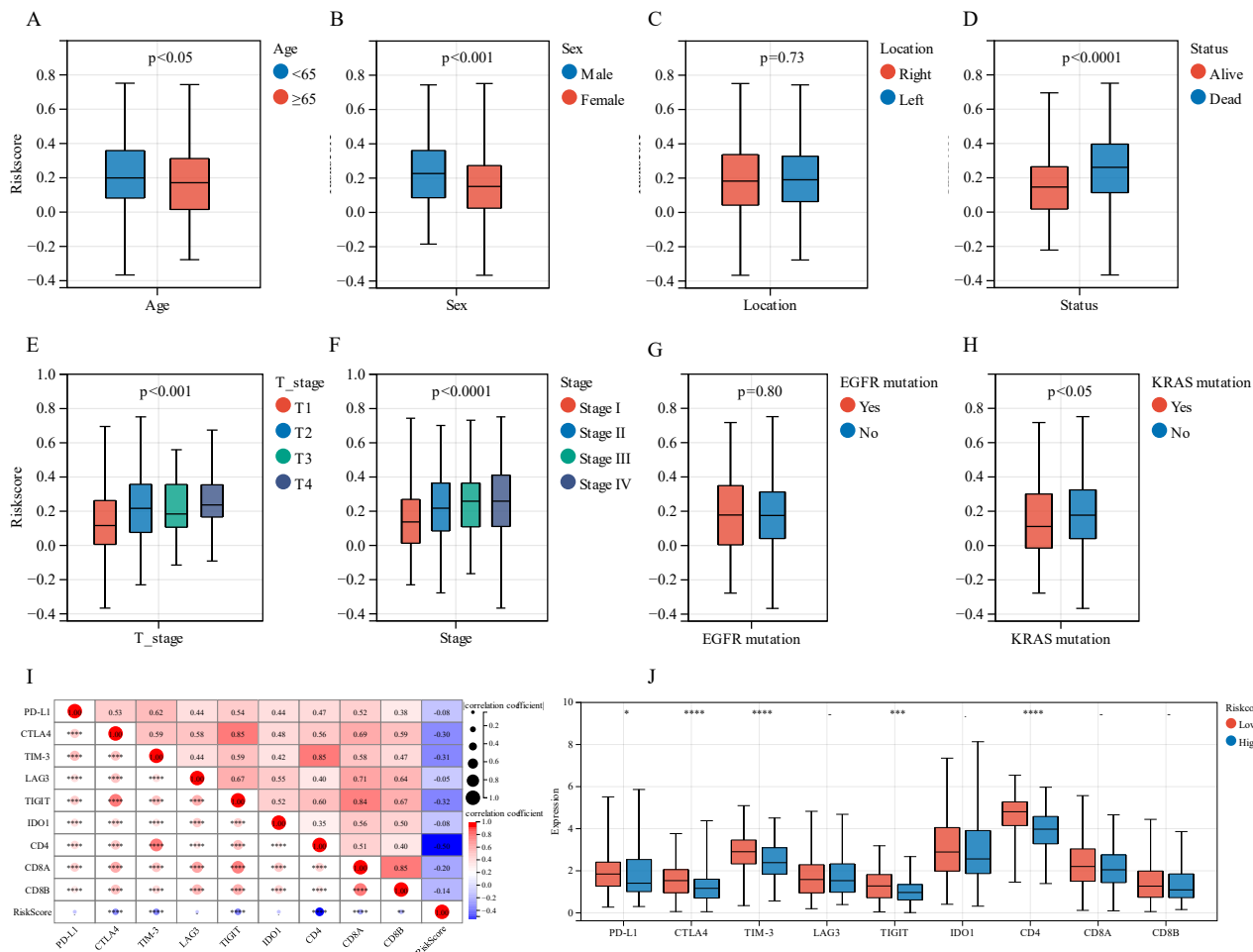

Supplement: Supplementary file 1 [file jcm-11-07223-s001.zip › Figure S4.pdf]

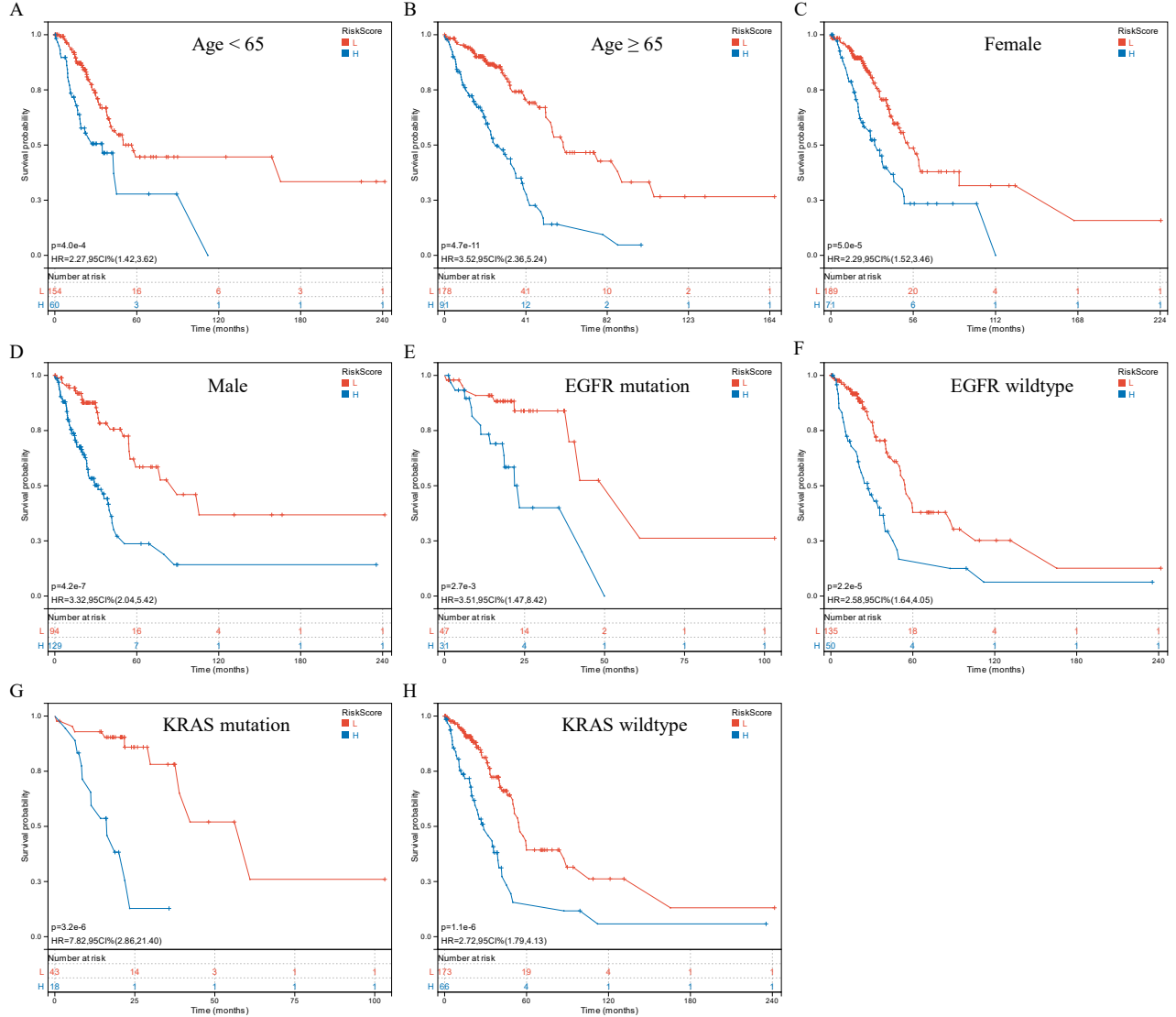

Supplement: Supplementary file 1 [file jcm-11-07223-s001.zip › Figure S5.pdf]
